# Neurokinin B Regulates Gonadotropin Secretion, Ovarian Follicle Growth, and the Timing of Ovulation in Healthy Women

**DOI:** 10.1210/jc.2017-01306

**Published:** 2017-10-12

**Authors:** Karolina Skorupskaite, Jyothis T. George, Johannes D. Veldhuis, Richard A. Anderson

**Affiliations:** 1 MRC Centre for Reproductive Health, The Queen’s Medical Research Institute, University of Edinburgh, Edinburgh EH16 4TJ, United Kingdom; 2 Warwick Medical School, Coventry CV4 7AL, United Kingdom; 3 Boehringer Ingelheim, Bracknell RG12 8YS, United Kingdom; 4 Endocrine Research Unit, Center for Translational Science Activities, Mayo Clinic, Rochester, Minnesota 55905

## Abstract

**Context:**

Neurokinin B (NKB) is obligate for human puberty, but its role in adult female gonadotropin secretion and ovarian follicle growth is unknown.

**Objective:**

To investigate antagonism of NKB on pulsatile gonadotropin-releasing hormone (GnRH) and luteinizing hormone (LH) secretion and ovarian follicle development in healthy women.

**Design:**

Open investigation of the effects of a neurokinin-3 receptor (NK3R) antagonist (NK3Ra) vs a no-treatment control cycle.

**Setting:**

Clinical research facility.

**Patients or other participants:**

Healthy women with regular menses (n = 13).

**Intervention(s):**

NK3Ra MLE4901 40 mg taken orally twice daily from cycle day 5 to 6 for 7 days.

**Main outcome measure(s):**

LH secretion, ovarian follicle growth, and timing of ovulation.

**Results:**

NK3Ra administration reduced basal LH secretion without a change in pulse frequency and delayed the LH surge by 7 days, the duration of treatment [mean cycle day ± standard error of the mean (SEM), 22 ± 1 days vs 15 ± 1 days in control cycles; *P* = 0.0006]. Follicle growth (mean diameter at the end of administration of NK3Ra administration ± SEM, 9.3 ± 0.4 mm vs 15.1 ± 0.9 mm in control cycles; *P* < 0.0001) and rising estradiol concentrations (mean ± SEM, 166 ± 29 pmol/L vs 446 ± 86 pmol/L in control cycles; *P* < 0.0001) were prevented. After treatment, follicle development resumed and normal preovulatory follicle diameter and estradiol concentrations were demonstrated. Postovulatory progesterone rise was similarly delayed (peak cycle day, 30 ± 2 vs 22 ± 1; *P* = 0.002) and cycle length was prolonged (35 ± 1 days vs 29 ± 1 days in control cycles; *P* = 0.0003) but luteal progesterone excretion was unaffected by the NK3Ra (LH surge day +7 mean urinary progesterone levels ± SEM, 58 ± 10 pmol/mol vs 48±7 pmol/mol creatinine in control cycles; nonsignificant).

**Conclusion:**

These data demonstrate the involvement of NKB-NK3R signaling in the physiological regulation of GnRH/LH secretion, determining normal follicle development in women.

## Teaser

We studied the role of NKB in human reproduction and found that NK3R antagonism suppresses basal GnRH/LH secretion and estradiol secretion, and postpones ovarian follicle growth in healthy women.

There is growing evidence that neurokinin B (NKB) is a key modulator of gonadotropin-releasing hormone (GnRH) secretion and, hence, of reproductive function in men and women. Loss-of-function mutations in the genes encoding NKB (*TAC3*) and the neurokinin-3 receptor (*TAC3R*) result in hypogonadotropic pubertal delay (1). This is similar to the phenotype of individuals with loss of function of kisspeptin signaling (2–4), and these neuropeptides can be coexpressed in some hypothalamic neurons (5). However, although kisspeptin administration results in stimulation of GnRH and luteinizing hormone (LH) secretion in men and women, albeit variably, according to the stage of the menstrual cycle and underpinning sex steroid environment (6–9), administration of NKB as an intravenous infusion over 3 hours to men and women had no effect on reproductive hormone secretion (10). Data from animal studies are also inconclusive; both stimulatory and inhibitory effects of NKB have been reported in rodent models (11–14). In higher species, a stimulatory effect of NKB on LH secretion has been more consistently shown, as demonstrated in ewes (15, 16), goats (17), and monkeys (18).

Administration of neurokinin-3 receptor (NK3R) antagonist (NK3Ra) has, however, provided evidence of the involvement of this pathway in human and animal reproduction through the regulation of GnRH secretion. When administrated to gonadectomized ewes, the NK3Ra ESN364 and MRK-08 decreased LH secretion and pulse frequency while follicle-stimulating hormone (FSH) levels were maintained (16, 19). Administration of ESN364 throughout the follicular phase in intact nonhuman primates inhibited estradiol secretion, with no LH surge or subsequent rise in serum progesterone level (19). Similarly, the NK3Ra MLE4901 (formerly known as AZD4901) resulted in a decrease in LH concentrations with a reduction in LH pulse frequency in healthy women during estrogen administration (20) and in women with polycystic ovary syndrome (PCOS) (21). In healthy women, ESN364 administration for 21 days from early in the follicular phase decreased estradiol secretion, although no significant changes in LH concentrations or follicle development were observed (22). The LH surge, however, was variably delayed (22). In an analysis of the interaction with kisspeptin in the regulation of surge-like LH secretion in health women, MLE4901 shortened the duration of kisspeptin-stimulated LH secretion (20).

We have explored, therefore, the role of NKB signaling in the regulation of GnRH and LH secretion and ovarian function in healthy women using the NK3Ra MLE4901. The effects of NK3R antagonism on LH secretion and ovarian follicle development during the follicular phase of the menstrual cycle were determined in premenopausal women, demonstrating that NKB-NK3R signaling is important in the physiological regulation of GnRH-driven follicle growth and, consequently, the timing of ovulation.

## Materials and Methods

### Participants

Thirteen healthy, premenopausal women, aged 27 to 41 years and with regular menstrual cycles (25 to 34 days) were recruited into the study; all provided informed written consent. Mean ± standard error of the mean body mass index was 26.6 ± 1.6 kg/m^2^. The women were not taking any hormonal contraception nor had an intrauterine device *in situ*. They had normal physical examination and full blood cell count results; renal function, electrolyte levels, liver function, and electrocardiogram results were within normal limits.

### Sample size

The sample size in this and previous studies is based on similar proof-of-concept mechanistic studies in our laboratory and by other colleagues, demonstrating significant changes in reproductive hormone concentrations and in pulsatile LH secretion (7, 9, 23). For LH pulse frequency, power analysis [based on data reported by Skorupskaite et al (20)] indicates that with *α* = 0.05, eight women per group would be required to show a significant change with 90% power.

### Study drug

The NK3Ra MLE4901 was administered orally at 40 mg twice daily. This dosage of MLE4901 reduced LH secretion in healthy women and in women with PCOS (20, 21).

### Protocol

#### Investigation of the effect of NK3R antagonism on gonadotropin and ovarian hormone secretion

**Figure 1.**
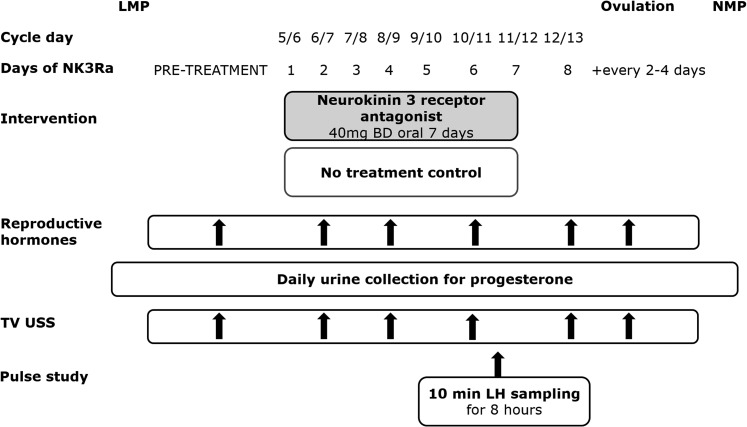
Study protocol schematic. The NK3Ra MLE4901 was administered orally to 13 healthy women for 7 days starting on cycle day 5 to 6. Reproductive hormones were measured and TV USS was performed pretreatment, on days 2, 4, 6, and 8 during the study, and then every 2 to 4 days until ovulation. Urine samples were collected daily until the next menstrual period. LH pulsatility (n = 8) was assessed during 8 hours of blood sampling every 10 minutes on day 6 or 7 of NK3Ra administration or on the equivalent day of the control cycle. Reproductive hormones and ultrasound scan findings were compared with those of the control cycle, the order of which was randomized. BD, twice daily; LMP, last menstrual period; NMP, next menstrual period; TV USS, transvaginal ultrasonography.

Schematic presentation of the protocol is shown in Fig. 1. All women had a treatment cycle and a no-treatment control cycle, with a washout cycle in between if the treatment cycle was first; the order of cycles was randomized using sealed envelopes. In the treatment cycle, women were administered the specific NK3Ra MLE4901 orally at 40 mg twice daily for 7 days starting on cycle day 5 to 6. Peripheral venous blood was sampled for LH, FSH, inhibin B, and estradiol concentration measurement between 08:00 and 10:00 immediately before treatment (pretreatment), and on days 2, 4, and 6 of NK3Ra administration immediately before the next dose of NK3Ra was taken (*i.e.*, 12 hours after the previous dose), in the morning after the last dose, and then every 2 to 4 days until ovulation was confirmed by transvaginal ultrasonography (Xario 200, 7.5 MHz probe frequency; Toshiba, Zoetermeer, The Netherlands). Ovulation was defined either by the last day on which the preovulatory follicle was seen or the appearance of a corpus luteum. In the control cycle, blood sampling and ultrasound timing were equivalent to that in the treatment cycle. A first-void specimen of urine was collected daily throughout the cycle.

#### Investigation of the effect of NK3R antagonism on follicle development and endometrial thickness

Transvaginal ultrasonography was used to measure the diameter of the leading follicle, any follicles ≥10 mm in diameter, endometrial thickness, and appearance of corpus luteum. Ultrasound scans were performed on the same days as assessment of reproductive hormone levels.

#### Investigation of the effect of NK3R antagonism on LH pulsatility

Assessment of LH pulsatility was performed in eight of the 13 women (owing to the time commitment, not all women volunteered for this) on day 6 or 7 of NK3Ra treatment or on cycle day 10 to 12 (the equivalent day) of control cycles. All visits commenced between 08:00 and 09:00. The dose of NK3Ra was administered when the pulsatility assessment was begun; blood samples were collected via an indwelling intravenous cannula at 10-minute intervals for 8 hours.

### Safety monitoring

Hematological and biochemical safety monitoring was performed before commencing NK3Ra treatment, at end of drug administration, and 2 to 3 weeks later.

### Hormone assays

Blood samples were centrifuged at 4°C for 10 minutes at 3000 rpm and serum was frozen at −20°C or below until analysis. LH and FSH levels were measured by enzyme-linked immunosorbent assay (ELISA) as previously described (23) with interassay and intra-assay coefficient of variation (CV) <5% at the concentrations measured. 17*β*-estradiol was measured on a Roche Cobas E411 immunoassay automated analyzer (Roche Diagnostics, Burgess Hill, United Kingdom). The lower limit of quantification was 18.4 pmol/L. The inter- and intra-assay CVs were <5% and 6.5%, respectively. Inhibin B was measured by ELISA (Beckman Coulter, Brae, CA) with a limit of quantification 2.6 pg/mL and intra-assay CV <8%; all samples were assayed in one run.

Progesterone level was measured by an inhouse ELISA. The interassay CVs for low and high progesterone pools, respectively, were 11.4% and 9.1%, and the respective intra-assay CVs were 8.9% and 5.6%. The lower limit of detection was 0.1 ng/mL. Urinary progesterone concentrations were expressed as a ratio of the creatinine concentration, measured colorimetrically (Alpha Laboratories, Eastleigh, United Kingdom), and adapted for use on a Cobas Fara centrifugal analyzer (Roche Diagnostics, Welwyn Garden City, United Kingdom). Intra-assay CV was <3%; all samples were analyzed in one batch, in duplicate.

The number of LH pulses, secretory mass of LH per pulse, and basal (nonpulsatile) and pulsatile (integral of dual amplitude and frequency regulation) LH secretion were identified by an established deconvolutional algorithm with cluster analysis (93% sensitivity and specificity) blinded to treatment allocation, and approximate entropy was quantified as a measure of secretory regularity (24, 25).

### Statistical analysis

Baseline characteristics between the control and NK3Ra-treated cycles were compared by Student paired *t* test (for normally distributed data, *i.e.*, LH, FSH, and estradiol levels; and follicle size) or Wilcoxon matched-pairs signed-rank test (menstrual cycle day). Serum hormone concentrations and ultrasonography data were compared throughout 7 days of NK3Ra treatment and between the control and treatment groups using repeated measure two-way analysis of variance (ANOVA) followed by Bonferroni multiple comparisons *post hoc* analysis. Peak serum hormone concentration, size of the preovulatory follicle, and cycle timing were compared by Student paired *t* test. Urinary progesterone concentrations were compared by two-way ANOVA followed by Bonferroni *post hoc* multiple comparisons test; these data were available for 11 women. Midluteal (*i.e.*, LH surge +7 days) urinary progesterone levels and cycle length were compared by Student *t* test. Characteristics of LH pulsatile secretion were compared by paired Student *t* test.

Data are presented as mean ± standard error of the mean. Data not normally distributed were log-transformed before statistical analysis. Differences were regarded as significant at a two-sided *P* < 0.05. The statistical software package GraphPad Prism 7 (GraphPad Software, San Diego, CA) was used.

### Ethical approval

The study protocol was approved by South East Scotland Research Ethics Committee (Reference 09/S1101/67). This study was formally assessed as not being a clinical trial of an investigational medicinal product and, therefore, was not linked to trials databases.

## Results

### Baseline characteristics

Each woman took part in control and treatment cycles, which were comparable by cycle day on which the study had started; baseline serum LH, FSH and estradiol concentrations; the size of the largest ovarian follicle present at that time; and the cycle day for assessment of LH pulsatility (Table 1).

**Table 1.** **Baseline Characteristics of Study Participants (n = 13)**

Characteristic	Control Cycle*^a^*	NK3Ra Cycle*^a^*	*P* Value
Cycle day study commenced	5.6 ± 0.2	5.0 ± 0.2	0.06
LH, IU	4.9 ± 0.5	4.8 ± 0.4	0.98
FSH, IU	5.1 ± 0.8	5.3 ± 0.9	0.82
Estradiol, pmol/L	160 ± 19	126 ± 13	0.07
Follicle diameter, mm	8.4 ± 0.4	8.1 ± 0.3	0.54
Cycle-day pulsatility study	11.1 ± 0.5	10.9 ± 0.2	0.75
LH pulses/h at baseline	0.69 ± 0.1	N/A	N/A

Data given as mean ± SEM.

Abbreviation: N/A, not applicable.

^a^ Control and NK3Ra-treated cycles were comparable by starting cycle day, reproductive hormone levels, and the size of the largest ovarian follicle.

### Basal and pulsatile gonadotropin secretion

**Figure 2.**
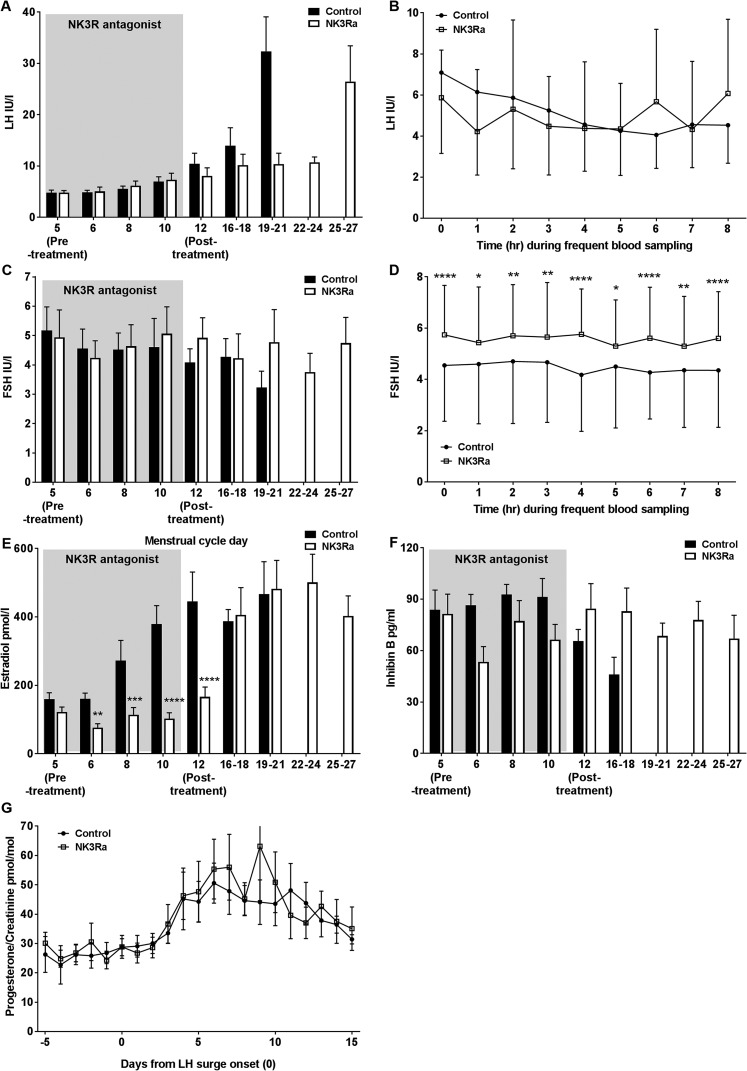
Reproductive hormone response in premenopausal women in the control and NK3Ra-treated cycles (n = 13). Mean serum (A) LH level during single timepoint sampling and (B) during 8 hours of frequent sampling every 10 minutes on day 6 to 7 of NK3Ra administration. (C) FSH level during single timepoint sampling and (D) during 8 hours of frequent sampling every hour on day 6 to 7 of NK3Ra administration. (E) Estradiol, (F) inhibin B secretion, and (G) urinary progesterone/creatinine ratio in premenopausal women (n = 11) adjusted to LH surge onset day 0 with and without NK3Ra was compared in premenopausal women by repeated measure two-way ANOVA at time points when paired data were available, followed by Bonferroni multiple comparisons *post hoc* analysis. Data are given as mean ± SEM. *P < 0.05; **P < 0.01; ***P < 0.001; ****P < 0.0001.

**Figure 3.**
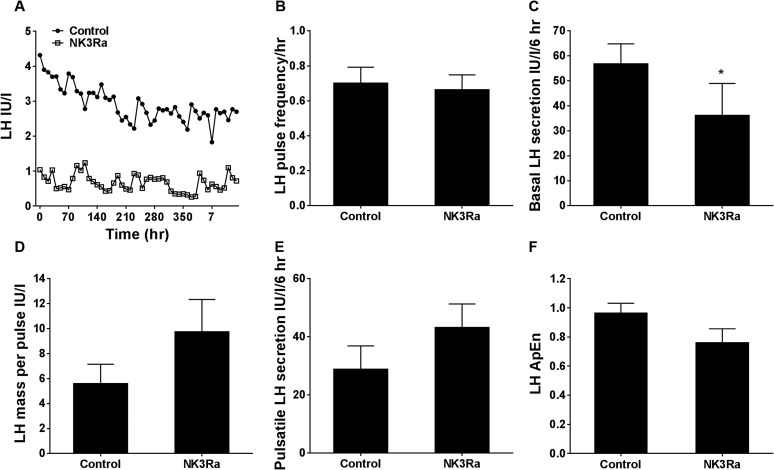
Pulsatile LH secretion in premenopausal women in the control and NK3Ra-treated cycles (n = 8). (A) Illustrative LH pulse profile from one participant who underwent blood sampling every 10 minutes for LH for 8 hours with no NK3Ra (closed circles) and on day 7 of NK3Ra treatment (open squares). Mean (B) LH pulse frequency, (C) basal (nonpulsatile) LH secretion, (D) mass of LH per pulse, (E) pulsatile LH secretion, and (F) relative orderliness or regularity of LH secretory pattern were compared between the control and NK3Ra-treated cycles. Data are given as mean ± SEM. *P < 0.05. ApEn, approximate entropy.

NK3R antagonism had no effect on LH secretion by single timepoint analysis throughout 7 days of administration (Fig. 2A) or by hourly analysis during 8 hours of frequent LH sampling after dosing on day 6 or 7 of NK3Ra treatment and on the equivalent day of the control cycle (Fig. 2B). However, deconvolutional analysis of pulsatile LH secretion, performed in eight of the 13 women on day 6 or 7 of NK3Ra treatment (cycle day 10.9 ± 0.2) and on the equivalent day of the control cycle (cycle day 11.1 ± 0.5), showed differences in pulsatile LH secretion between treatment and control cycles. An example of an LH pulse frequency profile is shown in Fig. 3A and the pulse profile for each participant is summarized in Supplemental Table 1. LH pulse frequency did not change with NK3Ra treatment [0.69 ± 0.1 pulses/h vs 0.66 ± 0.1 pulses/h in control cycles; nonsignificant (ns)], but basal (*i.e.*, nonpulsatile) LH secretion was reduced (*P* < 0.05; Fig. 3B and 3C). NK3Ra had no effect on secretory mass of LH per pulse (Fig. 3D) and total amount of LH secreted in a pulsatile manner (Fig. 3E). A slight increase in the orderliness (*i.e.,* decrease in the approximate entropy) of LH secretory pattern with the NK3Ra approached statistical significance (*P* = 0.054; Fig. 3F).

**Figure 4.**
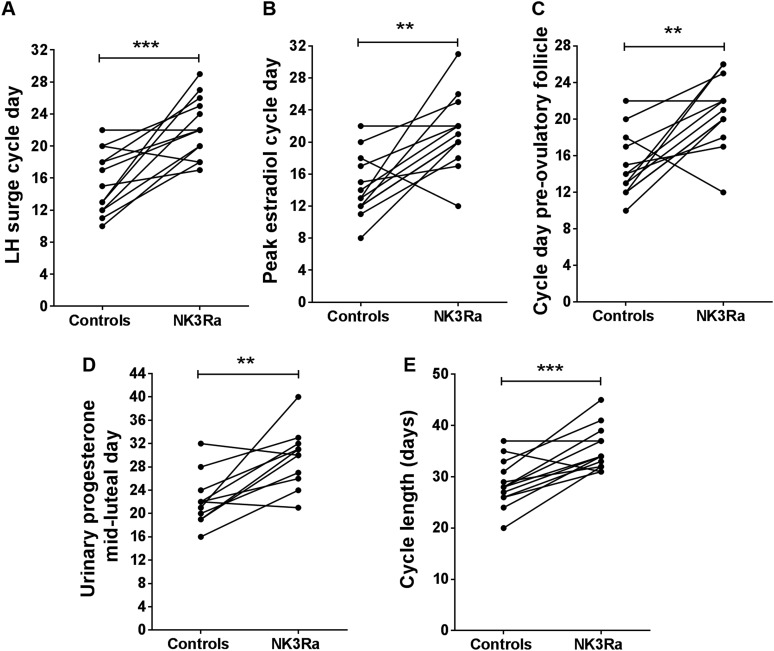
Summary of the delay in timing of key events in female reproduction with the NK3Ra compared with no-treatment control cycles in premenopausal women. Individual response of the participants (n = 13) to NK3Ra showing the day of the menstrual cycle for (A) LH surge, (B) peak estradiol, (C) appearance of the largest diameter of the preovulatory follicle, (D) midluteal urinary progesterone (day of LH surge +7; n = 11), and (E) the length of menstrual cycle. **P < 0.01; ***P < 0.001.

After discontinuation of MLE4901 treatment, an LH surge was detected on cycle day 22 ± 1 vs 15 ± 1 in control cycles (*P* = 0.0006; Fig. 2A and Fig. 4A). There was no effect on the magnitude of the peak of midcycle LH secretion (23.4 ± 4.8 IU/L vs 19.7 ± 3.2 IU/L in control cycles; ns).

FSH secretion was unchanged with the NK3Ra by single-day sampling (Fig. 2C). To detect subtle changes in hormone secretion over time potentially overlooked by performing single-time spot blood sampling, a more detailed analysis of FSH secretion every hour for 8 hours after NK3Ra dosing showed higher FSH concentrations throughout the 8-hours during NK3Ra administration compared with control cycles (ANOVA *P* < 0.03 and *P* < 0.05 for NK3Ra cycle vs control cycles at every hour; Fig. 2D).

### Follicular phase estradiol and inhibin B secretion

Serum concentrations of estradiol were affected by NK3Ra administration (Fig. 2E). Estradiol concentrations were significantly lower at each time point throughout treatment (*P* < 0.05 vs control cycles) and, at the end of NK3Ra administration, estradiol concentrations were markedly lower than in control cycles (166 ± 29 pmol/L vs 446 ± 86 pmol/L, respectively, on day 12; *P* < 0.0001), remaining comparable to baseline levels on cycle day 5 (166 ± 29 pmol/L at end of treatment vs 122 ± 14 pmol/L on day 5; ns). Over the days after discontinuation of NK3Ra treatment, estradiol concentrations rose, reaching preovulatory levels comparable those in control cycles (690 ± 68 pmol/L vs 699 ± 62 pmol/L, respectively; ns) but 7 days later (cycle day 21 ± 1 vs 14 ± 1, respectively; *P* = 0.002; Fig. 4B). Inhibin B concentrations were slightly reduced during NK3Ra administration, but this did not reach statistical significance (Fig. 2F).

### Follicle growth, the timing of ovulation, and endometrial development

**Figure 5.**
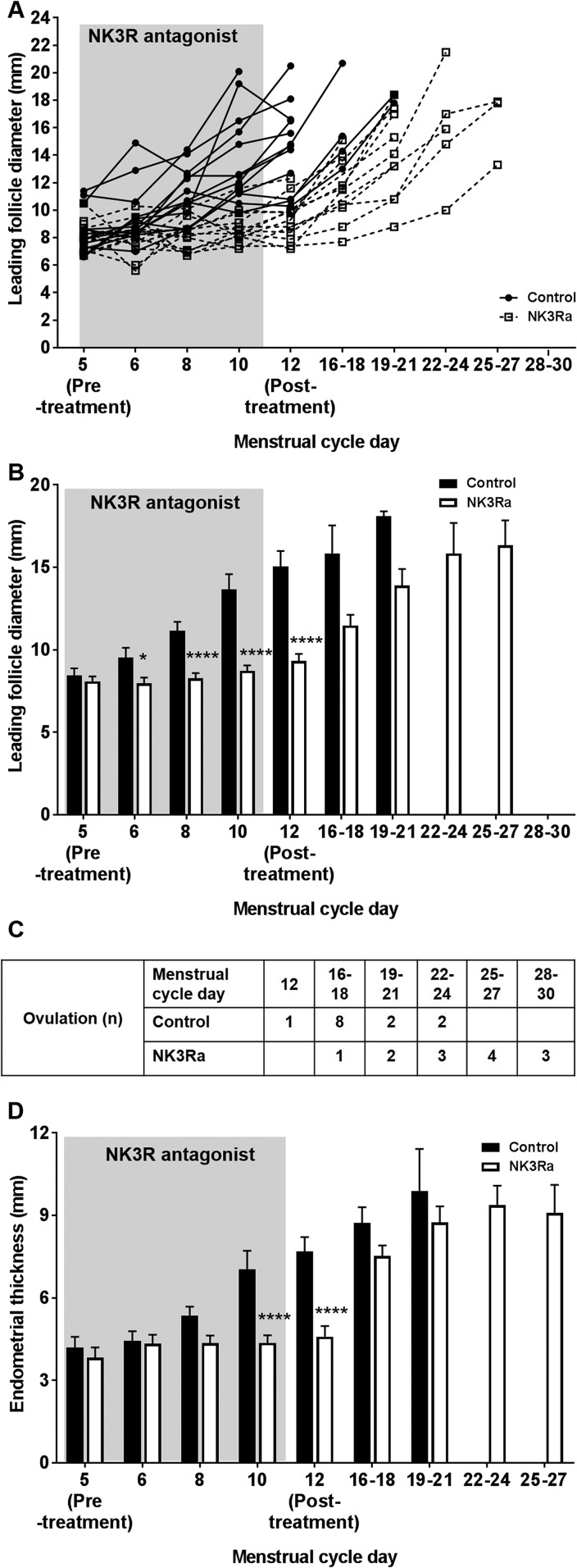
Follicle and endometrial development in 13 premenopausal women in the control and NK3Ra-treated cycles until ovulation. (A) Follicle growth in each of the 13 premenopausal women showed delayed development with the NK3Ra treatment (open squares) compared with the control cycle (closed circles). (B) Mean follicle diameter in the control and NK3Ra-treated cycle. Data include all cycles in which the leading follicle remained (*i.e.*, data only include women who had not yet ovulated at the later time points). (C) Table showing the time point in the control and NK3Ra cycle at which the leading follicle was no longer identified at transvaginal ultrasonography. (D) Endometrial development in premenopausal women in control and NK3Ra-treated cycles. Data are given as mean ± SEM. *P < 0.05; ****P < 0.0001.

Follicle growth was suppressed in NK3Ra treatment cycles, matching the effects on estradiol secretion. Figure 5A shows follicle growth until ovulation for each woman in the control and NK3Ra-treated cycles; the mean data are shown in Fig. 5B. Although there was a progressive rise in diameter of the leading follicle in control cycles, this did not occur during the 7 days of NK3Ra treatment (*P* = 0.0003). The diameter of the leading follicle was significantly smaller than in control cycles at each time point throughout the 7 days of treatment (*P* < 0.05 vs pretreatment) and at the end of treatment with the NK3Ra [*i.e.*, on cycle day 12 (9.3 ± 0.4 mm vs 15.1 ± 0.9 mm; *P* < 0.0001; Fig. 5B)]. After treatment, normal follicle growth resumed, reaching the same preovulatory follicle size as in control cycles (16.1 ± 0.7 mm with NK3Ra vs 17.2±0.7 mm in control cycles; ns) but later (cycle day 21 ± 1 vs 15 ± 1; *P* = 0.002; Fig. 4C). Consistent with the delay in the timing of the LH surge, NK3Ra treatment delayed the ultrasound-determined day of ovulation in 11 of 13 subjects (Fig. 5C).

Endometrial development was also affected by NK3Ra treatment (*P* < 0.0001); the endometrium was significantly thinner than in control cycles at the end of treatment (4.6 ± 0.4 mm vs 7.7 ± 0.5 mm, respectively, on day 12; *P* < 0.0001; Fig. 5D). Thereafter, endometrial thickness increased, reaching a similar thickness to that in control cycles at the time of ovulation (8.9 ± 0.6 mm vs 9.5 ± 1.0 mm, respectively; ns).

### Luteal progesterone secretion and cycle length

Consistent with the demonstration of delayed ovulation, NK3Ra treatment delayed the cycle day of peak midluteal progesterone (cycle day 30 ± 2 vs 22 ± 1; *P* = 0.002; Fig. 4D). However, when standardized against the day of the LH surge, luteal function was not affected by the NK3Ra (urinary progesterone level, 58±10 pmol/mol vs 48±7 pmol/mol creatinine on LH surge day +7; ns; Fig. 2G). Menstrual cycle length was prolonged, on average, by 6 days in the NK3Ra treatment cycle (35 ± 1 days vs 29 ± 1 days; *P* = 0.0003; Fig. 4E).

### Tolerability and safety

MLE4901 was well tolerated with no treatment discontinuations. Hematology and biochemistry (including liver function) safety parameters remained stable in all participants throughout the study (Supplemental Table 2). No participants had elevated liver function parameters related to drug administration. One woman’s bilirubin level was 1.4-fold higher than the upper range of laboratory reference pretreatment (within the limits of protocol allowance) with no change during treatment or thereafter. All participants returned to their usual menstrual cycle length after NK3Ra treatment.

## Discussion

This study investigated the role of the NKB pathway in regulating physiological follicle development and its hypothalamic regulation through the modulation of pulsatile GnRH and LH secretion in healthy women. This period of the cycle includes emergence of the dominant follicle and its growth toward ovulation, and thus is critical for female cyclicity and fertility. NK3R antagonism for 7 days in the early follicular phase in healthy women suppressed follicle growth and estradiol secretion, and delayed ovulation by the duration of treatment. NK3Ra suppressed basal LH secretion without a change in pulse frequency, detected by frequent blood sampling following drug administration, but LH concentration by once-daily sampling 12 hours after the previous NK3Ra dose was unchanged. The half-life of MLE4901 is approximately 8.5 hours (26). FSH secretion was increased during treatment, likely reflecting the reduced estradiol concentrations. FSH secretion is also promoted (relative to LH) by basal rather than pulsatile GnRH secretion (27), thus two mechanisms may contribute to the raised FSH concentrations observed with the NK3Ra. These findings demonstrate that selective blockade of NK3R regulates ovarian function by reducing basal GnRH and LH secretion in the midfollicular phase, and this effect persisted for the duration of treatment. This confirms an important role of NKB in human reproduction and, furthermore, provides evidence for NKB-NK3R signaling in the physiological regulation of normal follicle development in women.

A striking finding was that the effects of the NK3Ra were reversible after discontinuation of treatment, with normal follicular estradiol production and growth resuming, resulting in a normal LH surge and midluteal progesterone rise, all of which were delayed by the duration of treatment. Thus, although basal LH secretion was reduced during treatment, there remained sufficient gonadotropin support to the emerging dominant follicle to prevent atresia, although whether oocyte quality might have been compromised is unclear.

The decreased estradiol secretion for the duration of the 7 days of treatment was biologically relevant, as shown by lack of endometrial development during NK3Ra treatment, with subsequent growth to normal preovulatory thickness as follicle estradiol production increased after drug discontinuation. A different NK3Ra (ESN364) administered to healthy women for 21 days throughout the follicular phase did not result in any significant suppression in follicle growth, despite other findings being consistent with our data, particularly a delayed LH surge in some women and prolongation of menstrual cycle length (22). It is likely that the more variable effect in that study between different women precluded clear demonstration of an effect on folliculogenesis. Although these data show that dominant follicle development was delayed by treatment with the NK3Ra, there was no clear evidence of an effect on the growth of smaller follicles, as indicated by nonsignificant changes in inhibin B levels.

The recovery of LH and FSH concentrations 12 hours after dosing may indicate that the dose and regimen used in this study are at the bottom of the dose-response curve. Similarly, ovulation was delayed in 11 of the 13 women, and two women were nonresponders. It is possible that with higher doses of NK3Ra and longer duration of exposure, a more marked inhibitory action on LH secretion would be observed, potentially leading to follicle atresia and a decline in estradiol concentrations. Conversely, although follicle growth was arrested during 7 days of NK3Ra treatment, it remains unclear if such effect would persist with longer use. Nevertheless, consistent with suppressive effects on basal LH secretion during frequent blood sampling, antagonism of NK3R resulted in marked ovarian effects: Follicle maturation and ovarian hormone secretion were delayed, postponing the LH surge and subsequent luteal progesterone rise. The lower serum estradiol levels observed with the NK3Ra treatment suggests that LH dependent secretion of thecal androgens was suppressed. Granulosa cell proliferation (and, thus, follicle growth) is predominantly driven by FSH, which was not suppressed but rather increased by the NK3Ra. It may be that the NK3Ra, therefore, impaired follicle function through direct intrafollicular mechanisms, because NK3R has been localized to human granulosa cells (28–30). The results of this study, however, are in contrast to the suppressive effects of GnRH antagonists, and of the kisspeptin analog TAK448 (31), which, like GnRH agonists, resulted in an initial stimulation of LH secretion followed by suppression. Estradiol levels remained >100 pmol/L in this study. This alleviates concerns of unwanted menopausal-like adverse effects, which are associated with the use of GnRH analogs (32). Selective blockage of NKB signaling, therefore, might have therapeutic potential in the management of sex-steroid dependent disorders, such as endometriosis, fibroids, and heavy menstrual bleeding, and potentially as a nonsteroidal contraceptive.

This study investigated in detail a hypothalamic mode of action of NKB during the follicular phase of the menstrual cycle in healthy women, showing that, in the follicular phase, NK3Ra reduced basal LH levels (*i.e.*, the amount of LH secreted between the pulses) and, by inference, GnRH secretion, with no effect on pulse frequency. This indicates that basal rather than pulsatile LH secretion may be more important in supporting midfollicular phase follicle growth, with pulsatile secretion and frequency becoming more important in the lead up to ovulation, as occurs physiologically (33). We have recently demonstrated that the dose of MLE4901 used in this study reduced LH pulse frequency and abolished the correlation between estradiol and LH response to kisspeptin in a model of estrogen-induced LH secretion (20), which is in contrast to the absence of effect on LH pulse frequency observed in the current study in women with lower estradiol during early follicular phase. This suggests that estrogen feedback may have a role in modulating NKB effects on GnRH and LH secretion. The absence of effect on LH pulse frequency shown in the current study may reflect lower estradiol feedback (25), but may also reflect differences in the settings of the pathways driving GnRH pulse frequency in the early follicular phase. Reduction in the frequency of LH and GnRH pulses by the NK3Ra has been demonstrated previously in states of high LH output, in women with PCOS (21) and in gonadectomized ewes and male monkeys (16, 19). Patients with inactivating mutations in the NKB pathway had diminished LH pulse frequency (34); therefore, it remains possible that with larger doses of the NK3Ra, such an effect would be observed in healthy women in the follicular phase of the menstrual cycle. This study may also be underpowered to detect changes in LH pulse frequency.

In summary, NK3R antagonism in healthy premenopausal women in the early and midfollicular phase of the menstrual cycle resulted in reduced LH secretion, prevented follicle growth and rising estradiol secretion, and delayed ovulation by the duration of treatment. Those effects were reversible after cessation of drug administration, with evidence of normal ovulation and luteal function. These data confirm the involvement of NKB in the physiological neuroendocrine control of female reproduction, with potential therapeutic application in the management of sex steroid–dependent disorders.
