# Supplementary material for: Neurokinin B Regulates Gonadotropin Secretion, Ovarian Follicle Growth, and the Timing of Ovulation in Healthy Women
Source: J Clin Endocrinol Metab. 2017 Oct 12;103(1):95–104. doi: 10.1210/jc.2017-01306 (PMC5761486; doi:10.1210/jc.2017-01306)
Supplement: Supplementary file 1 [file jc.2017-01306.st1.pdf]

Supplemental material

Subject	Pulses/hr		Basal secretion IU/l/6hr		Mass per pulse IU/l		Pulsatile secretion IU/l/6hr		ApEn	
	Control	NK3Ra	Control	NK3Ra	Control	NK3Ra	Control	NK3Ra	Control	NK3Ra
**1**	0.5	0.6	45.7	50.8	2.3	9.1	9.1	45.3	1.1	0.7
**2**	0.5	0.8	90.1	30.1	14.4	6.1	57.5	36.5	0.9	0.9
**3**	0.3	0.4	31.5	22.0	4.6	26.3	9.1	78.8	0.7	0.5
**4**	0.6	0.5	44.4	12.1	5.6	7.5	28.1	29.9	0.7	0.7
**5**	1.0	1.0	92.4	40.9	2.7	7.5	21.8	59.8	1.2	1.1
**6**	0.8	1.0	38.3	4.0	3.3	1.1	19.5	8.8	1.0	1.1
**7**	0.9	0.6	54.5	117.4	10.0	12.4	70.2	62.2	1.1	0.7
**8**	1.0	0.4	57.1	11.5	1.9	7.9	14.9	23.7	1.0	0.4

Supplemental table 1. Pulsatile LH secretion parameters for each women in the follicular phase of the menstrual cycle with and without NK3R antagonist.

Subject	Liver function parameter	Pre-treatment	End of treatment	2-3 weeks later
**1**	**Bilirubin**	6	11	8
	**ALT**	11	11	6
	**Alkaline phosphatase**	62	62	62
	**AST**	21	18	16
**2**	**Bilirubin**	8	6	8
	**ALT**	10	14	14
	**Alkaline phosphatase**	92	89	82
	**AST**	19	18	19
**3**	**Bilirubin**	8	10	5
	**ALT**	13	11	13
	**Alkaline phosphatase**	59	54	58
	**AST**	23	22	33
**4**	**Bilirubin**	9	9	8
	**ALT**	17	23	26
	**Alkaline phosphatase**	64	72	69
	**AST**	17	25	23

**5**	**Bilirubin**	9	10	14
	**ALT**	13	12	15
	**Alkaline phosphatase**	46	44	40
	**AST**	14	14	16
**6**	**Bilirubin**	10	8	10
	**ALT**	17	14	17
	**Alkaline phosphatase**	41	40	40
	**AST**	18	14	18
**7**	**Bilirubin**	5	7	5
	**ALT**	19	21	26
	**Alkaline phosphatase**	55	52	52
	**AST**	21	22	20
**8**	**Bilirubin**	30	25	27
	**ALT**	7	11	9
	**Alkaline phosphatase**	48	60	53
	**AST**	16	20	18
**9**	**Bilirubin**	7	4	3
	**ALT**	12	18	35
	**Alkaline phosphatase**	80	88	112
	**AST**	12	22	24

**10**	**Bilirubin**	12	No result, lab error	9
	**ALT**	16	No result, lab error	14
	**Alkaline phosphatase**	38	No result, lab error	47
	**AST**	20	16	24
**11**	**Bilirubin**	6	6	10
	**ALT**	9	10	10
	**Alkaline phosphatase**	92	79	70
	**AST**	19	18	19
**12**	**Bilirubin**	6	12	4
	**ALT**	23	23	20
	**Alkaline phosphatase**	59	55	52
	**AST**	18	20	16
**13**	**Bilirubin**	8	9	5
	**ALT**	18	20	17
	**Alkaline phosphatase**	61	63	75
	**AST**	19	22	23

Supplemental table 2. Liver function monitoring for each subject during NK3R antagonist administration taken pre-treatment, at the end of drug administration and 2-3 weeks later. Biochemistry reference values: bilirubin 3-21 μmol/l; alanine aminotransferase (ALT) 10-50

IU/l; alkaline phosphatase 40-125 IU/l; aspartate aminotransferase 10-45 IU/l. Shaded cells indicate liver function parameters above the upper range of laboratory values.
